# Potential clinical value of fibrinogen-like protein 1 as a serum biomarker for the identification of diabetic cardiomyopathy

**DOI:** 10.1038/s41598-024-57580-3

**Published:** 2024-05-05

**Authors:** Yao Liu, Min Wang, Jia-Bao Su, Xiao Fu, Guan-Li Zheng, Shan Guo, Li-Juan Zhang, Qing-Bo Lu

**Affiliations:** 1 Department of Ultrasound, The Fourth Affiliated Hospital of Nanjing Medical University, Nanjing, 210031 Jiangsu China; 2 Department of Basic Medicine, Wuxi School of Medicine, Jiangnan University, Wuxi, 214122 Jiangsu China; 3 Department of Anesthesiology, Affiliated Hospital of Jiangnan University, Jiangnan University, Wuxi, 214125 Jiangsu China; 4 Department of Endocrinology, Affiliated Hospital of Jiangnan University, Jiangnan University, Wuxi, 214125 Jiangsu China

**Keywords:** Cardiac function, Diabetic cardiomyopathy, Fibrinogen-like protein 1, Left ventricular ejection fraction, Type 2 diabetes mellitus, Diseases, Medical research, Risk factors

## Abstract

Diabetic individuals with diabetic cardiomyopathy (DbCM) present with abnormal myocardial structure and function. DbCM cannot be accurately diagnosed due to the lack of suitable diagnostic biomarkers. In this study, 171 eligible participants were divided into a healthy control (HC), type 2 diabetes mellitus (T2DM) patients without DbCM (T2DM), or DbCM group. Serum fibrinogen-like protein 1 (FGL-1) and other biochemical parameters were determined for all participants. Serum FGL-1 levels were significantly higher in patients with DbCM compared with those in the T2DM group and HCs. Serum FGL-1 levels were negatively correlated with left ventricular fractional shortening and left ventricular ejection fraction (LVEF) and positively correlated with left ventricular mass index in patients with DbCM after adjusting for age, sex and body mass index. Interaction of serum FGL-1 and triglyceride levels on LVEF was noted in patients with DbCM. A composite marker including serum FGL-1 and triglycerides could differentiate patients with DbCM from those with T2DM and HCs with an area under the curve of 0.773 and 0.789, respectively. Composite marker levels were negatively correlated with N-terminal B-type natriuretic peptide levels in patients with DbCM. Circulating FGL-1 may therefore be a valuable index reflecting cardiac functions in DbCM and to diagnose DbCM.

## Introduction

Diabetic cardiomyopathy (DbCM) is a chronic complication of diabetes that is defined as heart failure in the absence of other cardiac risk factors, such as hypertension, coronary artery disease, and valvular heart disease^1,2^. Systemic metabolic disorders (e.g. insulin resistance), subcellular component abnormalities (e.g. mitochondrial dysfunction), endothelial dysfunction, oxidative stress and dysfunctional immune modulation may be the potential pathological bases leading to DbCM in diabetes^3–5^. Generally, patients with DbCM exhibit presentations of significant diastolic and systolic impairment and left ventricular hypertrophy^6^. Although therapeutic approaches to treat DbCM, including controlling blood glucose and blood lipid levels^7^, are available, there is currently a lack of drugs that can effectively reverse myocardial injury. Peripheral biomarkers such as haemoglobin A1c (HbA1c), cardiac troponin I, adiponectin and inflammatory mediators may serve as feasible indicators for the early diagnosis of DbCM^8^, however, these are not specific biochemical markers to diagnose DbCM. As DbCM often has an asymptomatic presentation in the early stages, the identification of appropriate diagnostic biomarkers that can confirm DbCM in the early stages is an urgent requirement in a clinical setting.

Fibrinogen-like protein 1 (FGL-1) is a 68-kDa protein primarily secreted by the liver; it plays proliferation- and metabolism-related roles in physiological and pathological conditions^9,10^. Through stimulation by metabolic factors, e.g. hyperglycaemia and hyperlipidaemia, FGL-1 acts not only on hepatocytes but also on other tissues, such as adipose tissues and skeletal muscles to regulate insulin resistance^11^. Previous studies have shown that FGL-1 may mediate transforming growth factor-β1 signalling pathway–related protein synthesis in inducing fibrosis synthesis, further leading to endothelial dysfunction and vascular wall injury^12,13^. Thus, FGL-1 is related to the pathogenesis of vascular diseases such as aortic dissection and intracranial aneurysm^12,14^. Furthermore, peripheral FGL-1 levels in patients with type 2 diabetes mellitus (T2DM) are significantly increased compared with those in normal subjects, and the elevated FGL-1 is independently associated with fasting glucose levels, insulin resistance and impaired glucose tolerance^15,16^. As FGL-1 can affect vascular structure and function and be abnormally expressed in patients with T2DM, it is essential to investigate the possible role of FGL-1 in DbCM.

In the current study, we assessed changes in serum FGL-1 levels in patients with DbCM and analysed the potential relationships to parameters of cardiac functions and glucose and lipid metabolism. Moreover, we evaluated the diagnostic value of serum FGL-1 in DbCM.

## Results

### Clinical characteristics of participants

As shown in Table 1, there were no significant differences in age and sex among patients in the HC, T2DM and DbCM groups (all p > 0.05). Meanwhile, the body mass index (BMI) and results from cardiac ultrasonography showed significant differences among patients in the three groups (all p < 0.05; Table 1 and Supplemental Table 1).

**Table 1:** Clinical characteristics of participants.

	HC (n = 58)	T2DM (n = 58)	DbCM (n = 55)	P-value
Age (years)	70.97 ± 14.11	69.76 ± 15.29	71.29 ± 13.59	0.931*
Sex (male/female)	23/35	28/30	28/27	0.451^#^
BMI	23.70 ± 3.39	25.90 ± 3.26	24.85 ± 4.13	0.005^&^
Cardiac ultrasonography results				
FS (%)	35.91 ± 8.18	33.76 ± 1.92	18.92 ± 5.06	< 0.001*
LVEF (%)	63.81 ± 2.41	62.45 ± 2.39	39.07 ± 8.05	< 0.001*
LVMI (g/m^2^ BSA)	157.95 ± 90.64	160.05 ± 75.02	219.20 ± 113.20	< 0.001*
LVDd (mm)	45.09 ± 2.87	46.66 ± 3.61	54.93 ± 7.03	< 0.001*
IVST (mm)	10.03 ± 1.54	10.93 ± 1.49	10.71 ± 1.74	0.006*
LVPWT (mm)	9.90 ± 1.45	10.88 ± 1.58	10.65 ± 1.88	0.005*
Blood indices				
FGL-1 (ng/ml)	17.86 ± 7.57	20.96 ± 7.49	29.97 ± 15.54	< 0.001^&^
NT-proBNP × 10^4^ (ng/L)	–	–	1.12 ± 1.16	–
TC (mmol/L)	3.72 ± 0.69	3.67 ± 1.26	4.50 ± 1.40	0.001^&^
TG (mmol/L)	1.32 ± 0.67	1.19 ± 0.37	1.73 ± 0.95	0.002*
LDL-C (mmol/L)	2.57 ± 0.92	2.74 ± 1.03	2.84 ± 5.22	0.007*
HDL-C (mmol/L)	1.07 ± 0.22	1.12 ± 0.27	1.03 ± 0.27	0.236^&^
FBG (mmol/L)	5.07 ± 0.48	10.49 ± 3.23	9.52 ± 3.70	< 0.001*
HbAlc (%)	5.65 ± 0.46	9.32 ± 2.62	8.51 ± 1.97	< 0.001*
AST/ALT ratio	1.00 ± 0.30	1.04 ± 0.40	1.32 ± 0.64	0.008*

Data are presented as the mean ± standard deviation.

HC, healthy control; T2DM, type 2 diabetes mellitus; DbCM, diabetic cardiomyopathy, BMI, body mass index; FS, fractional shortening; LVEF, left ventricular ejection fraction; LVMI (g/m^2^ BSA), left ventricular mass index (normalised to body surface area); LVDd, left ventricular end-diastolic diameter; IVST: interventricular septal thickness; LVPWT: left ventricular posterior wall thickness; FGL-1, fibrinogen-like protein 1; TC, total cholesterol; TG, triglyceride; LDL-C, low-density lipoprotein cholesterol; HDL-C, high-density lipoprotein cholesterol; FBG, fasting blood glucose; HbAlc, haemoglobin A1c; AST/ALT ratio, aspartate transaminase-to-alanine aminotransferase ratio.

*P-value obtained using Kruskal–Wallis H test.

^&^P-value obtained using one-way analysis of variance.

^#^P-value obtained using Chi-square test.

**Figure 1:**
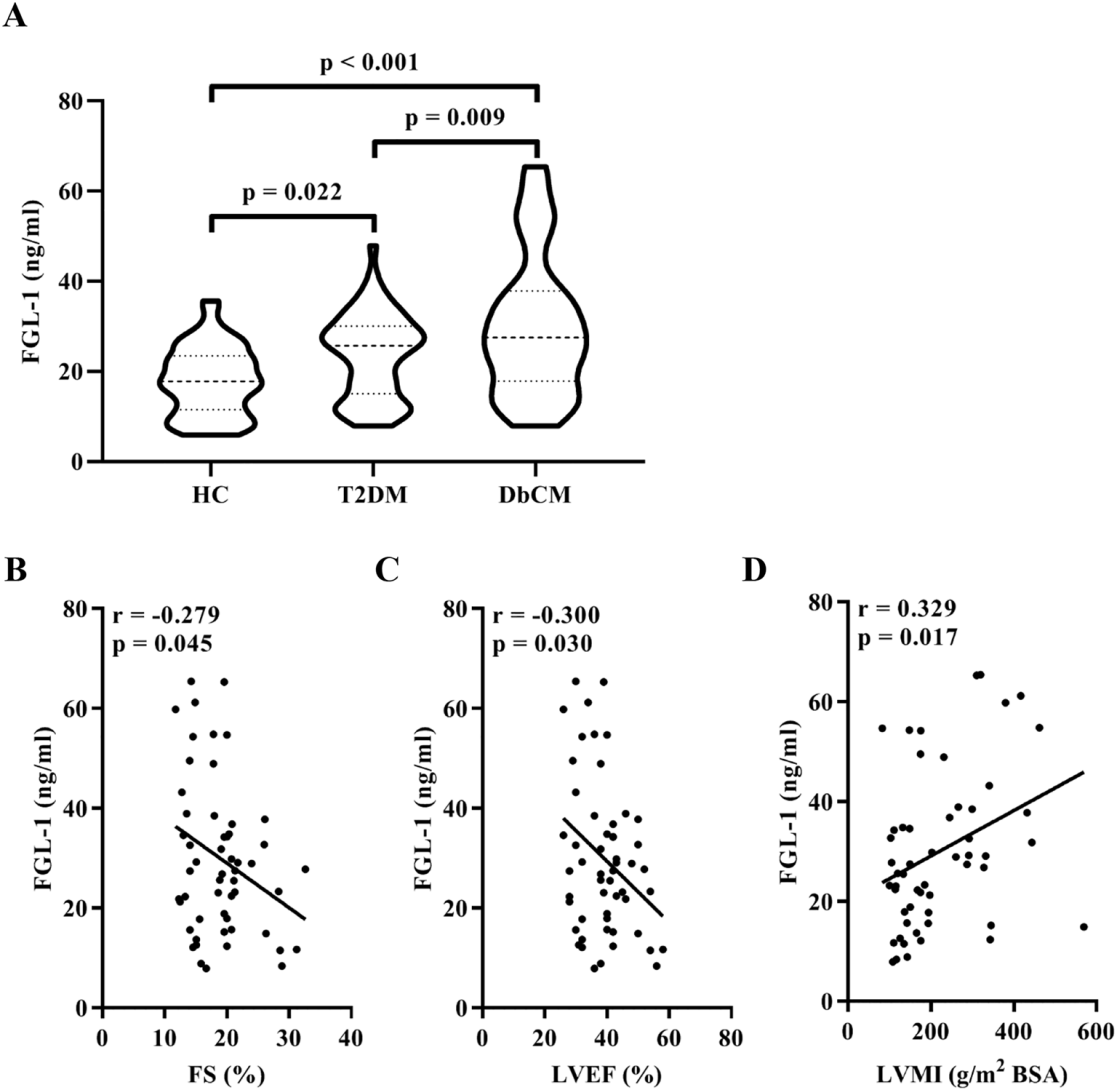
Analysis of serum FGL-1 levels. (**A**) Serum FGL-1 levels in the HC, T2DM, and DbCM groups. Bonferroni correction for post hoc test. (**B**–**D**) Correlations of serum FGL-1 levels with parameters of cardiac functions in patients with DbCM after adjusting for age, sex and BMI. Abbreviations: FGL-1, fibrinogen-like protein 1; HC, healthy control; T2DM, type 2 diabetes mellitus; DbCM, diabetic cardiomyopathy, BMI, body mass index; FS, fractional shortening; LVEF, left ventricular ejection fraction; LVMI (g/m^2^ BSA ), left ventricular mass index (normalised to body surface area).

Significant differences in the blood levels of total cholesterol (TC), triglyceride (TG), low-density lipoprotein cholesterol (LDL-C), fasting blood glucose (FBG), HbAlc and aspartate transaminase (AST)/alanine aminotransferase (ALT) (all p < 0.05; Table 1 and Supplemental Table 1) were observed among patients in the three groups; however, high-density lipoprotein cholesterol (HDL-C) levels were not significantly different (p < 0.05; Table 1 and Supplemental Table 1). As shown in Fig. 1A, patients with DbCM had significantly higher serum FGL-1 levels than those in the other groups, whereas patients with T2DM exhibited significantly increased serum FGL-1 levels compared with HCs.

### Association of serum FGL-1 with N-terminal B-type natriuretic peptide (NT-proBNP) and cardiac ultrasonography features of patients in the DbCM group

As shown in Fig. 1B–D, significant correlations were noted between serum FGL-1 levels and cardiac ultrasonography features after adjusting for age, sex and BMI. Among them, serum FGL-1 levels showed a significantly negative correlation with fractional shortening (FS) and left ventricular ejection fraction (LVEF) and a positive correlation with left ventricular mass index (LVMI) (normalised to body surface area). However, no significant correlation was found between serum FGL-1 and NT-proBNP levels (data not shown).

To comprehensively evaluate the association between serum FGL-1 and the occurrence of DbCM, a multivariate analysis followed by linear regression analysis was performed (Table 2). The analysis revealed that serum FGL-1 and TG levels were significantly associated with LVEF in patients with DbCM after adjusting for other confounders.

**Table 2:** Liner regression analysis to determine the association between clinical features and LVEFs of participants in the DbCM group.

	Standardised β	P-value
Age	0.149	0.247
Sex	0.082	0.534
BMI	−0.199	0.118
FGL-1	−0.303	0.020
TC	−0.010	0.951
TG	0.272	0.036
LDL-C	−0.175	0.170
HDL-C	0.012	0.923
FBG	0.131	0.308
HbAlc	0.172	0.175
AST/ALT ratio	−0.136	0.290

DbCM, diabetic cardiomyopathy; BMI, body mass index; FGL-1, fibrinogen-like protein 1; LVEF, left ventricular ejection fraction; TC, total cholesterol; TG, triglyceride; LDL-C, low-density lipoprotein cholesterol; HDL-C, high-density lipoprotein cholesterol; FBG, fasting blood glucose; HbAlc, haemoglobin A1c; AST/ALT ratio, aspartate transaminase-to- alanine aminotransferase ratio.

### Diagnosis of DbCM

**Figure 2:**
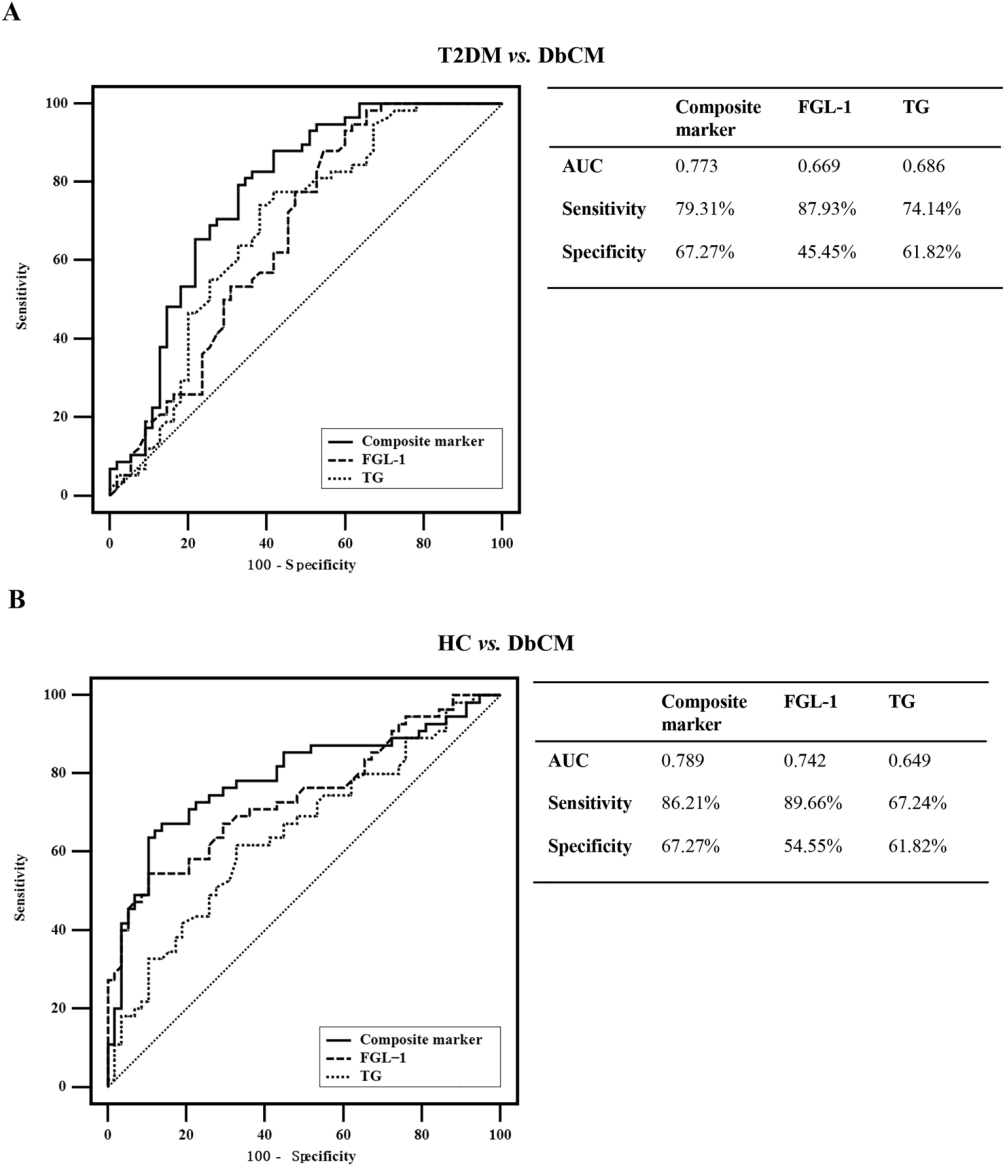
Receiver operating characteristic curve analyses. (**A**) Comparison of the diagnostic performance of 3 indices for differentiating between patients with DbCM and T2DM. (**B**) Comparison of the diagnostic performance of 3 indices for identifying DbCM individuals from HCs. Abbreviations: FGL-1, fibrinogen-like protein 1; HC, healthy control; T2DM, type 2 diabetes mellitus; DbCM, diabetic cardiomyopathy; AUC area under the curve.

Multivariate analysis revealed serum FGL-1 and TG to be potential biomarkers for the identification of patients with DbCM. Receiver operating characteristic (ROC) curve analysis revealed that serum FGL-1 or TG individually showed a non-ideal area under the curve (AUC) of < 0.7 in distinguishing patients with DbCM from those with T2DM (Fig. 2A), although the use of serum FGL-1 could differentiate patients with DbCM from HCs with an AUC of 0.742 (Fig. 2B).

**Figure 3:**
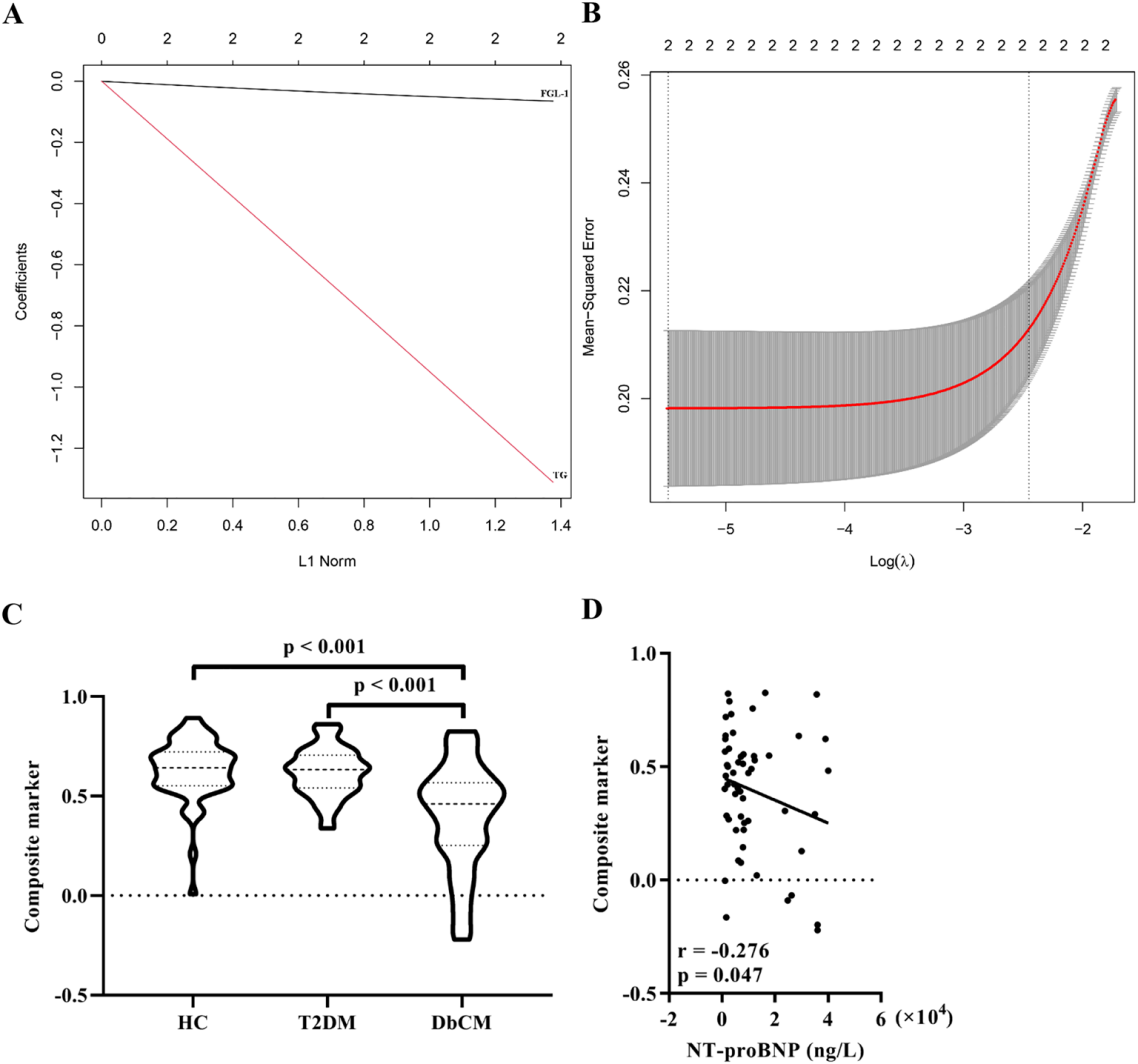
Construction and analyses of composite marker based on serum FGL-1 and TG levels. (**A**) Coefficients of 2 indices in the least absolute shrinkage and selection operator model. (**B**) Mean-squared error of model. (**C**) Significant differences in the levels of composite marker among the HC, T2DM and DbCM groups. Bonferroni correction for post hoc test. (**D**) Correlation analysis between levels of the composite marker and blood NT-proBNP levels in patients with DbCM. Abbreviations: FGL-1, fibrinogen-like protein 1; HC, healthy control; T2DM, type 2 diabetes mellitus; DbCM, diabetic cardiomyopathy; TG, triglyceride; NT-proBNP, N-terminal B-type natriuretic peptide.

To improve the accuracy in diagnosing DbCM, a composite diagnostic marker was built based on FGL-1 and TG indices using the least absolute shrinkage and selection operator (LASSO) model. The composite marker of the LASSO model was calculated as follows: composite marker = 1.12708925 − serum levels of FGL-1 × 0.01230558 – serum levels of TG × 0.20858438 (Fig. 3A,B). As shown in Fig. 2C, there was a significant difference in composite marker levels among patients in the HC, T2DM, and DbCM groups, and the levels of the composite marker in patients with DbCM were significantly reduced and had high specificity (Fig. 3C). Furthermore, there was a significantly negative correlation between the levels of the composite marker and NT-proBNP in patients with DbCM (Fig. 3D).

ROC curve analysis further indicated that the composite marker had an AUC of 0.773 in distinguishing patients with DbCM from those with T2DM (sensitivity = 79.31%, specificity = 67.27%; Fig. 2A). Moreover, the composite marker showed high accuracy in the differential diagnosis of patients with DbCM versus the HCs (AUC = 0.789, sensitivity = 86.21%, specificity = 67.27%; Fig. 2B).

## Discussion

The current study demonstrated the potential association between serum FGL-1 and DbCM. The main findings of this study were as follows: (1) Patients with DbCM had significantly higher serum FGL-1 levels than the HCs and patients with T2DM. (2) Cardiac ultrasonography revealed significant correlations between serum FGL-1 levels and FS, LVEF, and LVMI in patients with DbCM. (3) The interactive effect of serum FGL-1 and TG levels on LVEF was noted in patients with DbCM. (4) A composite marker including serum FGL-1 and TG showed a superior diagnostic power in distinguishing patients with DbCM from the HCs and patients with T2DM. Taken together, the novel biomarker FGL-1 played an important clinical role in mirroring the cardiac functions of DbCM and identifying patients with DbCM.

Fibrinogen plays a vital role in blood clotting. High levels of fibrinogen in the blood have been reported in vascular diseases including cardiovascular disease and T2DM^17–20^. FGL-1 is a member of the fibrinogen family that contains a fibrinogen domain at its C-terminal and shows high homology with fibrinogen^21^. As FGL-1 lacks the 3 functional domains of fibrinogen, namely, the platelet-binding site, the cross-linking region and the thrombin-sensitive site, it is irrelevant to the coagulation function^22^. Except in the liver, FGL-1 is found in brown and white adipose tissues and the bloodstream^23^. In this study, serum FGL-1 levels were significantly higher in patients with T2DM than in the HCs, which was consistent with findings from previous studies^15,16^. More importantly, significantly elevated serum FGL-1 levels were observed in patients with DbCM compared with the HCs and patients with T2DM, which suggested that FGL-1 might mediate the manifestation of cardiac function in T2DM. Further analyses indicated that serum FGL-1 levels were significantly correlated with FS, LVEF, and LVMI, as revealed during the cardiac ultrasonography of patients with DbCM. This finding suggested that serum FGL-1 could reflect the cardiac systolic and diastolic function of patients with DbCM. Although many studies have revealed that fibrinogen-like protein 2 is involved in the molecular mechanism of cardiomyocyte maturation and in the development of apoptosis in diabetes^24–26^, our findings provide new evidence on the underlying role of FGL-1 in cardiac function in T2DM.

Multivariate analysis indicated the interactive effect of serum FGL-1 and TG levels on LVEF in patients with DbCM, suggesting that abnormal lipid metabolism (mainly TG metabolism) might mediate the effect of FGL-1 on the myocardial perfusion and systolic function in DbCM. *Demchev *et al*.* found that FGL-1–deficient mice had abnormal plasma lipid profiles and showed differences in white and brown adipose tissue morphology compared with wild-type mice^27^, which supported that FGL-1 could regulate lipid metabolism. Furthermore, enhanced intramyocardial TG levels are strongly associated with DbCM^28^, and myocardial adipose triglyceride lipase protects diabetic mice from lipotoxic cardiomyopathy^29^. Therefore, the interaction of FGL-1 and TG may be an underlying pathological mechanism in the occurrence of DbCM.

Although FGL-1 demonstrated superiority in differentiating patients with DbCM from HCs, it was not satisfactory in distinguishing between patients with DbCM and those with T2DM. Based on previous findings, we combined the FGL-1 and TG indices to build a new composite marker in this study. Accordingly, using the LASSO model, a composite marker was obtained, which could provide an acceptable diagnostic performance to identify DbCM in either patients with T2DM or in HCs. The level of the composite marker showed a significant decrease in patients with DbCM versus that in the other two groups and was also not significantly different between the T2DM and HC groups, indicating that the marker was changed specifically in patients with DbCM. Additionally, in patients with DbCM, the composite marker level was significantly correlated with NT-proBNP level, further indicating that the marker was associated with the severity of cardiac functions. Thus, FGL-1 is an important biomarker for the diagnosis of DbCM. This finding was supported by the effective identification of the composite marker.

Our study has some limitations: (1) The dynamic changes in serum FGL-1 levels in patients with DbCM remain unclear, particularly in those who have transitioned from T2DM to DbCM. (2) The established composite marker could effectively identify DbCM in the present cohort; however, independent sample verification is necessary to further determine the generalisation of this composite marker in diagnosing DbCM. (3) The precise molecular mechanism of FGL-1 in DbCM could not be completely determined in this study. Animal models of diabetes will be used in our subsequent studies for elucidation of its mechanism. Accordingly, we will design a multi-centre study to verify our present findings and will use in vivo studies to determine the effect of FGL-1 in the pathophysiology of DbCM.

Overall, our study demonstrated that serum FGL-1 levels were significantly higher in patients with DbCM than in healthy subjects and in patients with T2DM. Moreover, circulating FGL-1 levels were significantly associated with the systolic and diastolic functions of the heart. In combination with serum TG levels, circulating FGL-1 levels can provide a superior diagnostic performance in identifying DbCM. Thus, in this study, we report the potential of the circulating biomarker FGL-1 in determining cardiac function and for the early diagnosis of DbCM, suggesting its role as a therapeutic target in diagnosing DbCM.

## Method and materials

### Participants

All participants or their legal guardians provided written informed consent, and the Ethics Committee of the Fourth Affiliated Hospital of Nanjing Medical University approved this study (approval number: 20180705-K048). All clinical investigations were performed in strict adherence to the principles outlined in the Declaration of Helsinki, and all experiments were performed in accordance with relevant guidelines and regulations.

A total of 113 patients of Chinese Han ethnicity with T2DM were recruited from the Fourth Affiliated Hospital of Nanjing Medical University (Nanjing, China). Additionally, 58 HCs were recruited from resident communities of Nanjing City. Each participant completed a standardised clinical interview, including a demographic inventory and examination of their physical and mental health. All participants underwent cardiac ultrasonography and blood collection.

According to the diagnostic criteria of the World Health Organisation in 1998^30^, patients with T2DM should have an FBG value ≥ 7.0 mmol/L, glucose levels ≥ 11.1 mmol/L after a 2-h post-glucose challenge, or both. Furthermore, patients with T2DM presenting with evidence of left ventricular diastolic dysfunction and without other cardiac diseases, such as coronary heart disease and hypertensive heart disease, were confirmed to have DbCM^31^. Next, patients with T2DM were divided into two groups, namely, the T2DM (n = 58) and DbCM (n = 55) groups.

The exclusion criteria were as follows: (1) Patients with hypertension, coronary heart disease or any other cardiovascular disease (such as arrhythmia, valvular disease, dilated cardiomyopathy); (2) patients with acute and serious chronic diabetic complications; (3) patients with a history of other comorbidities, such as severe hepatic, renal or thyroid dysfunction; tumour; trauma or any nervous system disease; (4) patients having organ infection (such as pulmonary infection) or other acute and chronic inflammatory diseases with significantly increased C-reactive protein levels or white blood cell counts; (5) women who were pregnant, lactating or climacteric.

### Clinical and biochemical evaluations

Echocardiography was conducted using a Vivid E9 ultrasound system (GE Healthcare, Milwaukee, WI, USA). The main parameters used in the present study included FS, LVEF, LVMI, left ventricular end-diastolic diameter, interventricular septal thickness and left ventricular posterior wall thickness.

After overnight fasting, the peripheral venous blood of patients was collected using a vacutainer tube (without any anticoagulant) and an EDTA-coated tube between 8:00 am and 9:00 am. Serum samples were obtained by centrifuging the collected samples at 3500 rpm for 10 min at 4 °C, and plasma samples were obtained by centrifuging the collected samples at 2000 × *g* for 10 min at 4 °C. All blood samples were stored at −80 °C until further use.

HbA1c levels of whole blood samples were measured using high-performance liquid chromatography (Agilent 1200 device, Agilent Technologies, Waldbronn, Germany). Serum TC, TG, LDL-C, HDL-C, FBG, AST and ALT levels were determined using a biochemical autoanalyser (UniCel DxC 600 Synchron, Beckman Coulter, California, United States). NT-proBNP in the plasma was measured using an electrochemiluminescence immunoassay (ProBNP Elecsys®, Roche Diagnostics GmbH).

### Detection of serum FGL-1

Erum FGL-1 levels were determined using a commercial enzyme-linked immunosorbent assay kit provided by Cusabio (CSB-EL008653HU, Houston, TX, USA)^32^. FGL-1 levels were measured in triplicate, with < 5% inter- and intra-assay coefficients of variation. No significant cross-reactivity or interference was observed.

### Statistical analyses

All statistical analyses were performed using SPSS version 22.0. Data are presented as mean ± standard deviation. Normal data distribution was confirmed using the Kolmogorov–Smirnov test. Several variables showed non-normal distribution and were analysed using Kruskal–Wallis H test for comparison of groups. One-way analysis of variance was used when other variables were normally distributed. Categorical variables were analysed using the Chi-square test. Interrelationships between variables were determined using partial correlation analysis after adjusting for age, sex and BMI. Multivariate linear regression analyses were used to analyse the association between FGL-1 and DbCM. The diagnostic accuracy of markers was determined using the ROC curve to obtain the AUC. The Youden index^33^ was used to estimate the optimal sensitivity and specificity values. P < 0.05 was considered statistically significant.

To obtain an approach for the clinical identification of DbCM, the LASSO model was used to construct a composite biomarker using R software (version 4.2.1) as used in previous studies^34,35^. LASSO regression can be used to remove trivial variables via regression coefficients penalising the size of the parameters^36^. LASSO regression, feature selection and predictive signature building were applied to construct a LASSO-derived composite biomarker model. The LASSO model is essentially a linear predictor of the binary model with selected variables via the LASSO algorithm^37^.

### Institutional review board statement

The Ethics Committee of the Fourth Affiliated Hospital of Nanjing Medical University approved the present study (Approval Number: 20180705-K048).

### Informed consent

A signed written informed consent form and acceptance to participate in the study were received from all participants in the study.

### Supplementary Information

Supplementary Information.

## Data Availability

The data supporting this study’s findings are available on request from the corresponding author.
