# Supplementary material for: Potential clinical value of fibrinogen-like protein 1 as a serum biomarker for the identification of diabetic cardiomyopathy
Source: Sci Rep. 2024 May 5;14:10311. doi: 10.1038/s41598-024-57580-3 (PMC11070422; doi:10.1038/s41598-024-57580-3)
Supplement: Supplementary file 1 — Supplementary Information. [file 41598_2024_57580_MOESM1_ESM.docx]

**Supplemental Table 1. Clinical characteristics of participants in the present study via post-hoc analysis.**

|  | HC *vs.* T2DM | DbCM *vs.* T2DM | DbCM *vs.* HC | F / H value |
| --- | --- | --- | --- | --- |
| Age (years) ^*^ | - | - | - | 0.14 |
| BMI^&^ | P=0.004 | P=0.368 | P=0.281 | 5.39 |
| Cardiac ultrasonography results | |  |  |  |
| FS (%)^*^ | P=0.141 | P<0.001 | P<0.001 | 113.07 |
| LVEF (%)^*^ | P=0.149 | P<0.001 | P<0.001 | 115.60 |
| LVMI (g/m^2^ BSA) ^*^ | P=1.000 | P=0.008 | P<0.001 | 16.91 |
| LVDd (mm) ^*^ | P=0.095 | P<0.001 | P<0.001 | 80.50 |
| IVST (mm) ^*^ | P=0.006 | P=1.000 | P=0.082 | 10.28 |
| LVPWT (mm) ^*^ | P=0.005 | P=1.000 | P=0.071 | 10.67 |
| Blood indices |  |  |  |  |
| TC (mmol/L) ^&^ | P=1.000 | P=0.001 | P=0.001 | 9.11 |
| TG (mmol/L) ^*^ | P=1.000 | P=0.002 | P=0.019 | 12.97 |
| LDL-C (mmol/L) ^*^ | P=1.000 | P=0.007 | P=0.060 | 9.95 |
| HDL-C (mmol/L) ^&^ | - | - | - | 1.46 |
| FBG (mmol/L) ^*^ | P<0.001 | P=0.420 | P<0.001 | 105.93 |
| HbAlc (%)^*^ | P<0.001 | P=0.878 | P<0.001 | 112.68 |
| AST/ALT ratio^*^ | P=1.000 | P=0.037 | P=0.013 | 9.62 |

^*^ P values were obtained using Kruskal–Wallis test with Dunn post hoc tests.

^&^ P values were obtained using One-way ANOVA test with Bonferroni post hoc test.
